# Tetrahydrobiopterin (BH4) deficiency is associated with augmented inflammation and microvascular degeneration in the retina

**DOI:** 10.1186/s12974-017-0955-x

**Published:** 2017-09-06

**Authors:** José Carlos Rivera, Baraa Noueihed, Ankush Madaan, Isabelle Lahaie, Jingyi Pan, Jaques Belik, Sylvain Chemtob

**Affiliations:** 1 Department of Ophthalmology, Maisonneuve-Rosemont Hospital Research Center, Université de Montréal, 5415 Blvd de l’Assomption, Montréal, Québec H1T 2M4 Canada; 2 Department of Pediatrics, Ophthalmology and Pharmacology, CHU Sainte-Justine Research Center, Université de Montréal, Montréal, QC, Canada; 3 Departments of Pediatrics and Physiology, The Hospital For Sick Children, University of Toronto, Toronto, Canada

**Keywords:** Tetrahydrobiopterin, BH4, Microvascular degeneration, Thrombospondin-1, Inflammation, Microglia

## Abstract

**Background:**

Tetrahydrobiopterin (BH4) is an essential cofactor in multiple metabolic processes and plays an essential role in maintaining the inflammatory and neurovascular homeostasis. In this study, we have investigated the deleterious effects of BH4 deficiency on retinal vasculature during development.

**Methods:**

*hph-1* mice, which display deficiency in BH4 synthesis, were used to characterize the inflammatory effects and the integrity of retinal microvasculature. BH4 levels in retinas from *hph-1* and wild type ﻿(WT)﻿ mice were measured by LC-MS/MS. Retinal microvascular area and microglial cells number were quantified in *hph-1* and WT mice at different ages. Retinal expression of pro-inflammatory, anti-angiogenic, and neuronal-derived factors was analyzed by qPCR. BH4 supplementation was evaluated in vitro, ex-vivo, and in vivo models.

**Results:**

Our findings demonstrated that BH4 levels in the retina from *hph-1* mice were significantly lower by ~ 90% at all ages analyzed compared to WT mice. Juvenile *hph-1* mice showed iris atrophy, persistent fetal vasculature, significant increase in the number of microglial cells (*p* < 0.01), as well as a marked degeneration of the retinal microvasculature. Retinal microvascular alterations in juvenile *hph-1* mice were associated with a decreased expression in Norrin (0.2-fold) and its receptor Frizzled-4 (FZD4; 0.51-fold), as well as with an augmented expression of pro-inflammatory factors such as IL-6 (3.2-fold), NRLP-3 (4.4-fold), IL-1β (8.6-fold), and the anti-angiogenic factor thrombospondin-1 (TSP-1; 17.5-fold). We found that TSP-1 derived from activated microglial cells is a factor responsible of inducing microvascular degeneration, but BH4 supplementation markedly prevented hyperoxia-induced microglial activation in vitro and microvascular injury in an ex-vivo model of microvascular angiogenesis and an in vivo model of oxygen-induced retinopathy (OIR).

**Conclusion:**

Our findings reveal that BH4 is a key cofactor in regulating the expression of inflammatory and anti-angiogenic factors that play an important function in the maintenance of retinal microvasculature.

**Electronic supplementary material:**

The online version of this article (10.1186/s12974-017-0955-x) contains supplementary material, which is available to authorized users.

## Background

Tetrahydrobiopterin (BH4) is a cofactor required by the aromatic amino acid hydroxylases enzymes to the phenylalanine metabolism and neurotransmitter biosynthesis [1]. BH4 is formed de novo from guanosine triphosphate (GTP), by the committing and rate-limiting enzyme GTP cyclohydrolase I (GTPCH I) [2]. BH4 acts as an essential cofactor for all nitric oxide synthase (NOS) isoforms and as such regulates for nitric oxide (NO) production [1]. Thus, GTPCH plays a major role in controlling NOS function [3].

BH4 is a metabolite that serves as a critical cofactor and inhibits superoxide generation from endothelial nitric oxide synthase (eNOS) [4]. Under normal physiological conditions, eNOS plays an important role in maintaining the healthy state of the endothelium. For instance, eNOs-derived NO plays a key role to maintain the vascular wall in a quiescent state by inhibition of inflammation, cellular proliferation, and thrombosis [5]. A deficiency in BH4 can cause the uncoupling of eNOS, leading to a reduction in NO bioavailability and increased reactive oxygen species (ROS) production [6].

Excessive ROS generation caused by BH4 deficiency has been associated with an augmented inflammatory response followed by endothelial dysfunction and tissue injury in a variety of vascular diseases [7, 8]. Several pro-inflammatory and growth factors have been reported to be associated with excessive oxidative stress generation thus causing deleterious effects on vasculature [8]. For instance, interleukin-1β (IL-1β), a major mediator of inflammation, has been implicated in vascular repulsion [9] and capillary degeneration [10, 11] in the eye. Under several insults including oxidant stress, microglial cells, the phagocytic sentinels in the retina become over-activated and function as a prominent source of IL-1β [10, 11], as well as another pro-inflammatory factors such as TNF-α, and IL-6 implicated in retinal microvascular injury [12–14].

Thrombospondin-1 (TSP1), a matricellular glycoprotein, is another important molecule secreted in response to injury and stress in the retina [15]. TSP1 is significantly increased in the vessels of type 2 diabetic rats [16] and endothelial progenitor cells from *hph-1* mice [17]. TSP-1 has a potent anti-angiogenic activity [18, 19], and mice deficient in TSP1 exhibit increased retinal vascular density [20]; meanwhile, its over expression results in the attenuation of retinal vascular development [21]. A previous study in our lab showed that trans-arachidonic acids generated in the retina during nitrative stress induce a TSP1-dependent microvascular degeneration [15]. In contrast, a recent study showed that GTPCH I overexpression, the rate-limiting enzyme of BH4 synthesis, rescues the regenerative capacity of diabetic-impaired endothelial progenitor cells by suppressing oxidative stress and TSP-1 levels [17].

Clinical evidences suggest potential benefits from BH4 supplementation by improving vascular function in patients with coronary artery diseases, hypertension, hypercholesterolemia, and diabetic vasculopathy [22–25]. Moreover, BH4 supplementation has shown a marked reduction in vascular dysfunction and infiltration of monocytes, T cells, and macrophages in animal models of atherosclerosis [26, 27].

Up to now, oxidative stress and inflammation have come to be regarded as important mechanisms involved retinal vascular diseases [11, 28, 29]. However, the effects of BH4 deficiency and inflammation in the eye have been poorly investigated. Recently it was demonstrated that in response to retinal hyperoxia, enhanced eNOS expression led to increased NOS-derived superoxide and dysfunctional NO production, nitrotyrosine accumulation, and exacerbated vessel closure associated with tetrahydrobiopterin (BH4) insufficiency [30]. In this study, we have used the *hph-1* mouse, which displays 90% deficiency in guanine triphosphate cyclohydrolase (GTP), the rate-limiting enzyme in BH4 synthesis to characterize the integrity of the retinal vessels, and the inflammatory effects of BH4 deficiency in the eye. Our results suggest that, after some days of BH4 deficiency, an excessive number of activated microglia produces an excess of pro-inflammatory factors such as interleukin-1β (IL-1β), NRLP-3, interleukin-6, as well angiostatic factors such as TSP1 and decreased expression of Norrin and its receptor Frizzled-4 (FZD4) that can be associated with retinal microvascular degeneration.

## Methods

### Animal care

*hph-1* mice were bred in-house and genotyped to confirm homozygous, dominantly inherited GTPCH1 gene deficiency (data not shown). C57BL/6 mice (Charles River, ON, Canada) were utilized as wild type (WT) controls, since this is the background of the *hph-1* mice utilized in this study. All experiments were conducted in accordance with ARVO statement regarding the use of animals in ophthalmic and vision research and approved by the Animal Care Committee of the Hôpital Maisonneuve-Rosemont and The Hospital for Sick Children in accordance with guidelines established by the Canadian Council on Animal Care.

### Histological analysis of the eyes from WT and *hph-1* mice

Eyes enucleated from WT and *hph-1* mice at postnatal day 1 (P1), P7, P14, and P22 were immediately immersed in a mixture of 2.5% glutaraldehyde and 4% paraformaldehyde for 24 h. Eyes were dehydrated and embedded in paraffin, and then, serial sections were cut ﻿at 10 μm thickness﻿﻿ with a microtome and examined to determine the center of optic nerve. Sections were stained with H&E and images obtained in an epifluorescence microscope (Zeiss AxioObserver.Z1) at 10×. Images were merged using the MosiaX option in the AxioVision 4.6.5 software.

### Real time quantitative PCR analysis.

Eyes were enucleated from WT and *hph-1* mice at postnatal day 7, 14, and 22, and retinas were rapidly dissected and processed for RNA using RiboZol (Amresco, N580, OH, USA). Total cellular RNA was isolated by acidic phenol/chloroform extraction followed by treatment with DNase I (Roche Diagnostics, Mannheim, Germany) to remove any contaminating genomic DNA. 1 μg of total RNA was reverse-transcribed into cDNA using iScript™ RT Supermix (BioRad) as described by manufacturer’s instructions. cDNA was analyzed by Quantitative real-time PCR using iTaq™ Universal SYBR® Green Supermix (BioRad) with primers targeting for "?>IL-1β; (5′- CTGGTACATCAGCACCTCACA-3′ and 5′-GAGCTCCTTAACATGCCCTG-3′), NRLP3; (5′-AGCCAGAGTGGAATGACACG-3′ and 5′-CGTGTAGCGACTGTTGAGGT-3′), IL-6; (5′-CACTTCACAAGTCGGAGGCT-3′ and 5′-CTGCAAGTGCATCATCGTTGT-3′), TSP-1; (5′- GCTGCCAATCATAACCAGCG-3′ and 5′-TTCGTTAAAGGCCGAGTGCT-3′), Norrin; (5′-CGCTGCATGAGACACCATTAT-3′ and 5′-CTCAGAGCGTGATGCCTGG-3′), Frizzled-4; (5′-TTCCTTTGTTCGGTTTATGTGCC-3′ and 5′- CTCTCAGGACTGGTTCACAGC-3′), Sema3A; (5′-GCTCCTGCTCCGTAGCCTGC-3′ and 5′- TCGGCGTTGCTTTCGGTCCC-3′) and Iba-1; (5′-CTGAGGAGCCATGAGCCAAA-3′ and 5′-CCAGCATTCGCTTCAAGGAC-3′). Primers were designed using Primer Bank and NCBI Primer Blast software. Quantitative analysis of gene expression was generated by using an ABI Prism 7500 Sequence Detection System and calculated relative to 18S universal primer pair (Ambion) expression using the ΔcT method.

### Retinal flat-mounts

Eyes were enucleated from WT mice and *hph-1* mice at P1, P7, P14, and P22 and fixed in 4% paraformaldehyde for 1 hour at room temperature and then stored in PBS at 4 °C until used. The cornea and lens were removed, and the retina was gently separated from the underlying choroid and sclera under a dissecting microscope. Then, the retinas were incubated overnight at 4 °C in 1% Triton X-100-1 mM CaCl_2_/phosphate-buffered saline (PBS) with the TRITC-conjugated lectin endothelial cell marker *Bandeiraea simplicifolia* (1:100; Sigma-Aldrich, St. Louis, MO), the specific microglia marker Iba-1 (1:500; Wako Chemicals USA, Inc], or the mouse monoclonal TSP-1 antibody (1:200; EMD Millipore). Iba-1 or TSP-1 antibodies were labeled for 2 h with Alexa-488-conjugated goat anti-rabbit (1:1000) or Alexa-594-conjugated goat anti-mouse (1:500), respectively, obtained from Molecular Probes (Eugene, OR). Retinas were washed in PBS and mounted on microscope slides (Bio Nuclear Diagnostics Inc., Toronto, ON) under cover slips with Fluoro-Gel® (Electron Microscopy Sciences, Hatfield, PA) as the mounting media. Retinas were photographed under a confocal microscope (Olympus, Richmond Hill, Canada) at 30× or an epifluorescence microscope at 10× using a Zeiss AxioObserver.Z1. Images were merged into a single file using the MosiaX option in the AxioVision 4.6.5 software (Zeiss). Vascular density was calculated for the full retina surface by using the software AngioTool as previously described [31]. AngioTool computes several morphological and spatial parameters including vascular density by assessing the variation in foreground and background pixel mass densities across an image [31].

### Immunofluorescence in retinal cryosections

Eyes were enucleated from WT and *hph-1* mice at P22 and then fixed in 4% paraformaldehyde at room temperature for 2 h. Eyes were incubated  overnight at 4 °C in a 30% sucrose solution prior to embedding in OCT compound (TissueTek®). Coronal sections of the eyes ﻿﻿were cut at a thickness﻿ ﻿of 10 μm by using a Cryostat (Leica). Sections were subsequently washed with PBS, blocked and permeabilized for 1 h at room temperature and subsequently incubated overnight with Lectin from *Bandeiraea simplicifolia* TRITC conjugate (Sigma; 1:100) for retinal vasculature and/or antibodies to rabbit Iba-1 (Wako Chemicals USA, Inc.; 1:400), goat IL-1β (R&D systems; 1:300), or rabbit anti-CD36 antibody (Abcam; 1:200). The primary antibodies were labeled for 2 h with Alexa-488 goat anti-rabbit or donkey anti-goat IgG obtained from Molecular Probes (Eugene, OR) and used at dilutions of 1:1000. Samples were visualized using 30× objective with an IX81 confocal microscope (Olympus), and images were obtained with Fluoview 3.1 software (Olympus).

### Tetrahydrobiopterin (BH4) content in retinal tissues

Retinal tissues (pool of five retinas per sample, *n* = 3 per group) were collected from WT mice and *hph-1* mice at postnatal day 7, 14, and 22. Determination of BH4 in retinal tissues was performed by liquid chromatography tandem mass spectrometry (LC-MS/MS) as previously described [32]. Briefly, analysis was performed on an AB Sciex 5500QTRAP mass spectrometer (Foster City, CA, USA) coupled with a Shimadzu Nexera ultrahigh pressure liquid chromatograph system (Kyoto, Japan). Pterins were separated by a binary gradient using reversed-phase HPLC on an EZfaast 250 × 2 mm 4 μm AAA-MS column, with a 4 × 2 mm SecurityGuard column. A calibration curve was prepared in water containing 0.2% dithiothreitol (DTT) (Sigma Aldrich, Oakville, ON) over the range of 25–1600 nmol/L for BH4. A deproteinization solution containing internal standards for ^15^N–BH4 was prepared in 0.1 M perchloric acid (Sigma Aldrich) containing 0.2% DTT at a final concentration of 1000 nmol/L each. Retinal tissue samples were deproteinized and then centrifuged at 14800 rpm at 4 °C for 10 min. Thirty microliter of supernatant was combined with 120 μl of water containing 0.2% DTT. Processed supernatants were transferred to a microtiter plate, and 10 μL was injected for analysis.

### Preparation of microglia condition media (MGCM)

Microglia cell line (SIM-A9) was used and cultured as previously reported [33]. Briefly, microglial cells (800, 000 cells per well) were cultured in 6-well plates (Sarstedt, Inc., Newton, NC, USA) with DMEM/F12 (1:1) supplemented with 10% fetal bovine serum (FBS), 5% of horse serum (HS), and 1% penicillin/streptomycin. After 24 h, the cells were starved with DMEM/F12 (1:1) free of FBS and HS for 6 h. Then, microglial cells cultures in presence or absence of 100 μM of (6R)-5,6,7,8-Tetrahydrobiopterin dihydrochloride (BH4; Sigma, Cat. T4425) were exposed to hyperoxia (75% oxygen and 25% nitrogen; Hyp-MGCM) in a modular incubator chamber (Billups-Rothenberg, Inc) and maintained in a humidified CO_2_ incubator at 37 °C for 24 h. Microglial cells in matching controls (Nor-MGCM) were incubated at 37 °C in an incubator with 95% air and 5% CO_2_ and collected at the same time point. Cell lysates were quickly processed for RNA using RiboZol (Amresco, N580, OH, USA). The conditioned media was stored at −80 and later used in choroidal explant assay.

### Choroidal sprouting assay

WT mice pups were sacrificed on P22, and eyes immediately enucleated and kept in ice-cold EBM-2 medium before dissection. Cornea and lens were removed from the anterior of the eye and choroid-scleral complex was separated from the retina and cut into approximately 1 mm × 1 mm. Choroid/sclera fragments were placed in 30 μL of growth factor-reduced BD Matrigel ^TM^ (BD Biosciences, Cat. 354,230) seeded in 24-well plates. After seeding the choroid, plates were incubated at 37 °C in a cell culture incubator without medium for 15 min for the BD Matrigel^TM^ to solidify. Five hundred microliters of EBM-2 medium (LONZA, Cat. CC-3156) supplemented with endothelium growth medium (EGM) kit (Lonza, Cat. CC-4147) and 50 units/mL of Penicillin/Streptomycin (GIBCO, Cat. 15,142) was then added to each well and incubated at 37 °C with 95% air and 5% CO_2_ for 48 h before any treatment. After this time, the media was removed and choroidal explants were incubated with Nor-MGCM or Hyp-MGCM in presence or absence of a TSP-1 neutralizing antibody (1.2 μg/ml; EMD Millipore) for 48 h. Phase contrast photos of individual explants were taken at days 4 and 6 using a ZEISS AxioOberver microscope, and the microvascular sprouting area was quantified with the computer software ImageJ 1.46r (National Institute of Health).

### Oxygen-induced retinopathy

Mice pups were exposed with their mothers in a 75% oxygen environment from postnatal day 7 to P9 using BioSpherix oxycycler (Model #A84XOV) to induce retinal vaso-obliteration (VO) as previously described [34, 35]. Animals were anesthetized in 3% isoflurane in oxygen and injected intravitreally at P7 with 100 μM of (6R)-5,6,7,8-Tetrahydrobiopterin dihydrochloride (BH4; Sigma, Cat. T4425) or vehicle (sterile PBS 1×) using a Hamilton syringe equipped with 50-gauge glass capillary. At P9, mice pups were sacrificed and retinas were dissected and stained overnight at 4 °C with fluorescein-labeled *Griffonia Simplicifolia* Lectin 1 (GSL 1), isolectin B4 (FL 1201, Vector Labs; 1:100) with 1 mM CaCl_2_ in PBS as described above. Quantification of VO was assessed using the computer software ImageJ as previously described [36].

### Statistical analysis

Results are expressed as mean ± SEM. Two-tailed independent Student *t* tests was used to analyze data after confirming that the data are normally distributed. Comparisons between groups were made using one-way ANOVA followed by the post hoc Bonferroni’s multiple comparison test. Statistical significance was set at *p* < 0.05.

## Results

### Morphological defects in the eyes of *hph-1* transgenic mice

**Fig. 1:**
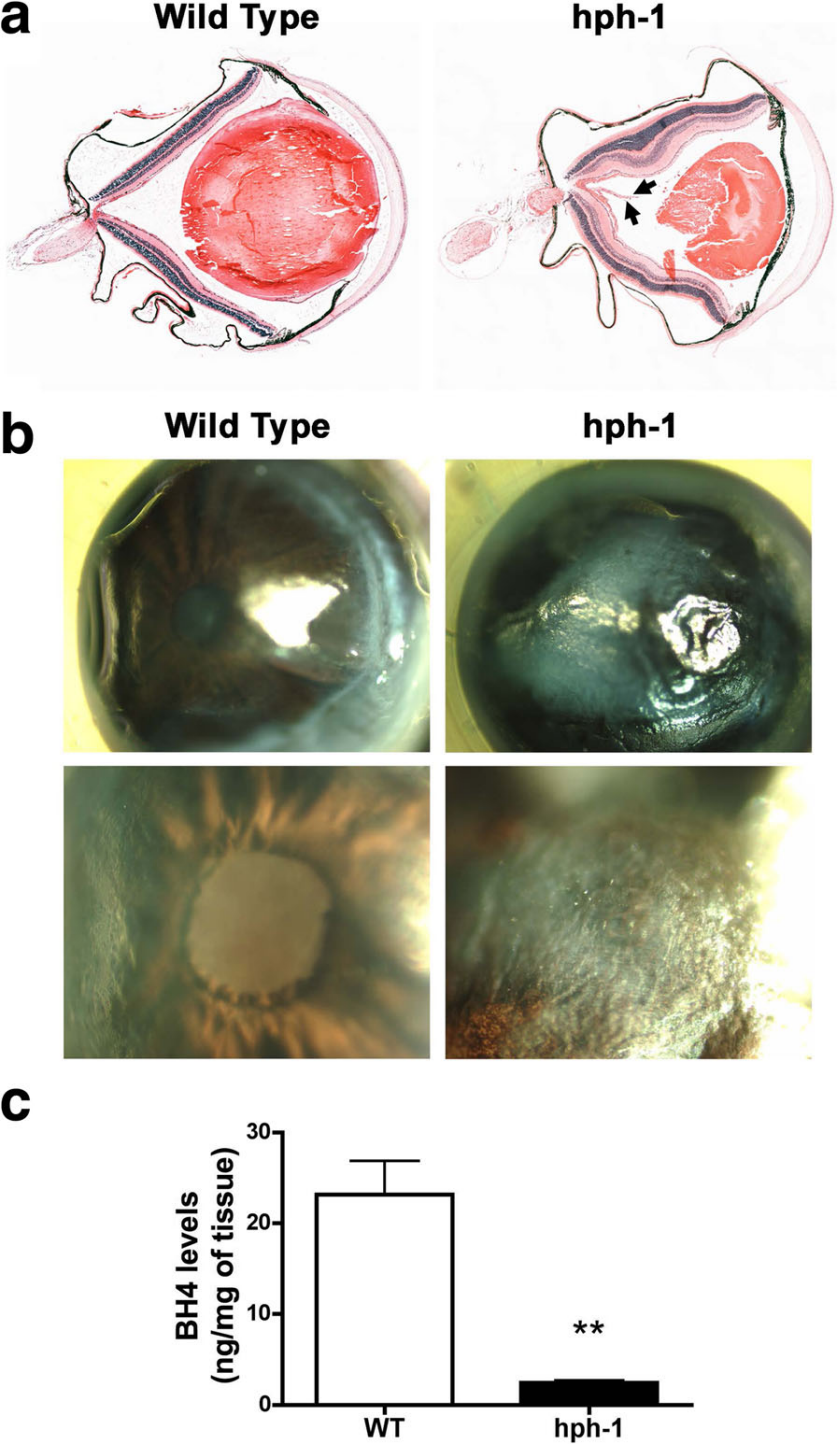
Tetrahydrobiopterin (BH4) deficiency is associated with morphological changes in the eye. **a** Representative images of the eyeballs from wild type (WT) and *hph-1* mice analyzed by H&E staining at postnatal day 22 (P22). The pictures show a reduction in the size, as well as, a persistence of fetal hyaloid vasculature in the eyes of *hph-1* (arrows). **b** Representative images of the anterior eyeball showing that the iris appeared hypertrophic, causing the pupil to be smaller or absent in an eye of hph-1 mice at P22, but not in WT. **c** BH4 content (ng/mg of tissue) in retinas from control (WT) and *hph-1* mice at P22 was measured by using liquid chromatography tandem mass spectrometry (LC-MS/MS). The levels of BH4 were significantly decreased by ~ 90% in the retinas of *hph-1* compared with WT mice. Results showed in the histograms are expressed as means ± SEM of *n* = 3 mice for each group. **p < 0.005

To evaluate whether BH4 deficiency is associated with morphological changes in the eye, histological analysis of eyeballs from WT and *hph-1* mice was analyzed by H&E staining at different post-natal ages (P1, P7, P14 and P22). Visual examination and histology revealed a decremented reduction in the size of the eyes from *hph-1* eye mice (at all the ages analyzed) compared to WT animals (Fig. 1a and Additional file 1: Figure S1). Also, in approximately 50% of the eyes from *hph-1* mice at P22, the iris appeared hypertrophic, causing the pupil to be smaller or absent (Fig. 1a, b). Persistence of fetal hyaloid vasculature was also detected in the eyes of *hph-1* at P22, but absent in WT mice with the same age (Fig. 1a; arrows).

### Tetrahydrobiopterin (BH4) levels are decreased in the retina of *hph-1* mice

To assess the levels of BH4 in the retina, from three to five pools of retinas were collected from WT and *hph-1* mice at postnatal age 7, 14, and 22 and evaluated by LC-MS/MS. LC-MS/MS analysis confirmed a significant decreased by approximately 90% in the concentration levels of BH4 in retinal tissue from *hph-1* mice (0.0009 ± 0.0006; *p* < 0.0001, 0.01 ± 0.001; *p* < 0.0001 and 2.45 ± 0.40; *p* < 0.005) compared to the WT group (0.014 ± 0.001, 0.092 ± 0.01, and 23.13 ± 6.44) at P7, P14, and P22, respectively (Fig. 1c and Additional file 2: Figure S2).

### Retinal microvascular degeneration is present in *hph-1* mice

**Fig. 2:**
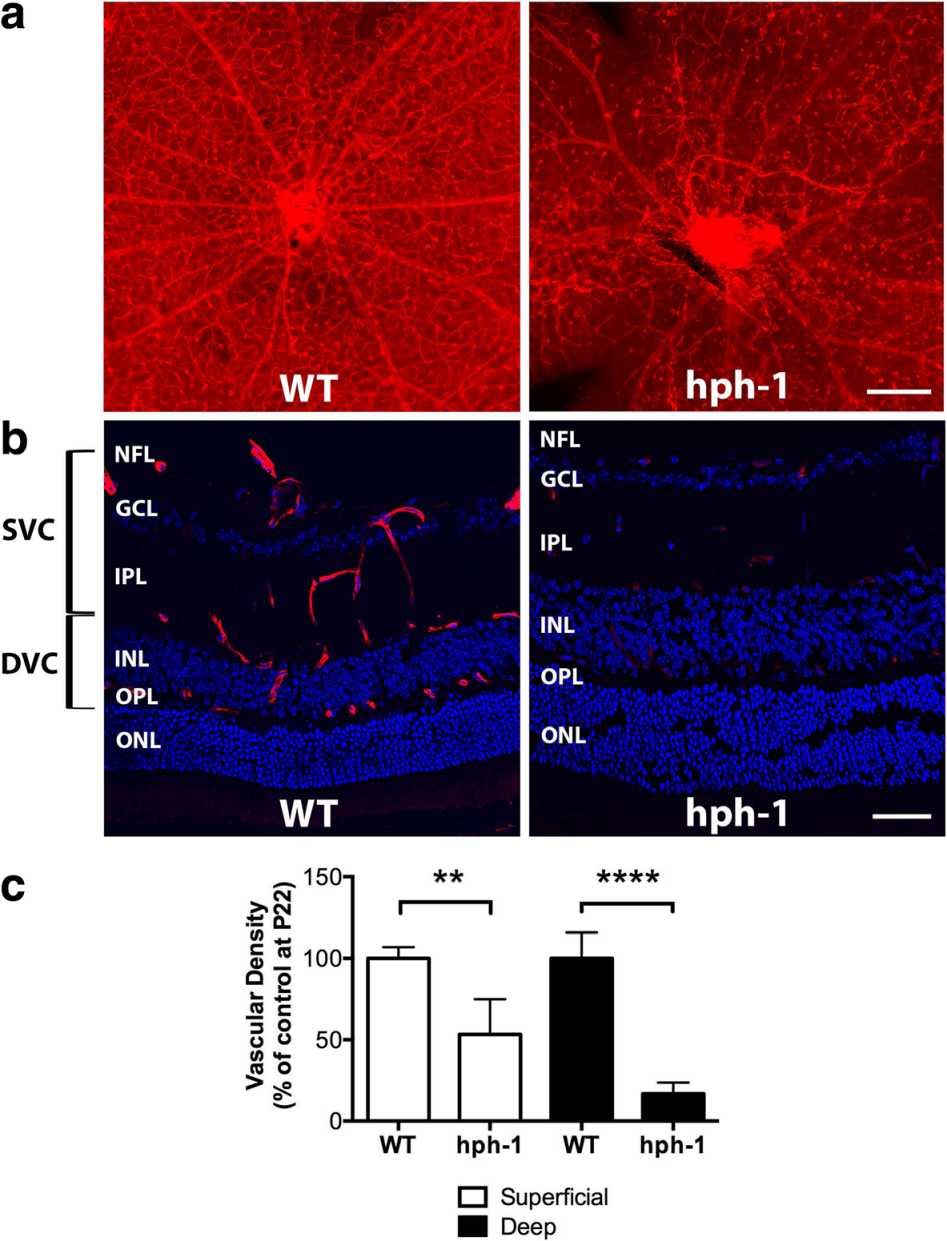
Tetrahydrobiopterin (BH4) deficiency is associated with retinal microvascular degeneration. **a** Representative images of whole-mounted retinas labeled with TRITC-conjugated lectin endothelial cell marker *Bandeiraea simplicifolia* showing microvascular degeneration in the superficial and deep retinal vascular complexes particularly noted in the central retina from *hph-1* animals at P22. Scale bar = 400 μm. **b** Representative confocal images of retinal cryosections labeled with TRITC-conjugated lectin (red) and DAPI (blue) showing the anatomic localization of the vascular complexes in the retinas from *hph-1* and WT mice. The vascular networks in the retina were grouped into superficial and deep vascular complexes (SVC and DVC) according to the current nomenclature [62]. NFL nerve fiber layer, GCL ganglion cell layer, IPL inner plexiform layer, OPL outer plexiform layer, and ONL outer nuclear layer. Scale bar = 50 μm. **c** The quantification of the vascular density in both superficial and deep retinal vascular complexes was significantly minor in *hph-1* mice compared to the WT control. Results showed in the histograms are expressed as means ± SEM of *n* = 3–4 retinas for each group. **p < 0.005, ****p < 0.0001 compared to the control

We further investigated whether a deficiency in BH4 synthesis could be associated with microvascular changes in the retina. Retinal whole-mounts from WT and *hph-1* mice at different postnatal days (P1, P7, P14, and P22) were analyzed (Fig. 2 and Additional file 3: Figure S3). In both groups of animals, the superficial retinal vascular sprouts were seen normally emerged from a ring-shaped vessel around the optic nerve head at P1 and reached the periphery at P7 (Additional file 3: Figure S3). At P14, the retinas from WT mice showed well-established superficial and deep vascular plexuses﻿ (Additional file 3: Figure S3)﻿, while the superficial and deep retinal vasculature from *hph-1* mice at P22 showed markedly signs of vascular injury particularly localized in the central retina (Fig. 2a, b). A quantitative analysis of the superficial and deep retinal microvasculature density in *hph-1* mice showed a significant reduction (*p* < 0.01 and *p* < 0.0001, respectively) in the number of vessels with respect to the control mice at P22 (Fig. 2c). However, no significant differences were detected in the density of both capillary plexuses in the retina from *hph-1* mice at early ages (P1, P7, and P14; Additional file 4: Figure S4). These results suggest that a constant deficiency in BH4 synthesis is associated with a degeneration of the microvasculature in the retina with the time and are consistent with previous studies demonstrating that BH4 is an important regulator of inflammation and vascular remodeling [37].

### Augmented number of inflammatory cells in *hph-1* mice

**Fig. 3:**
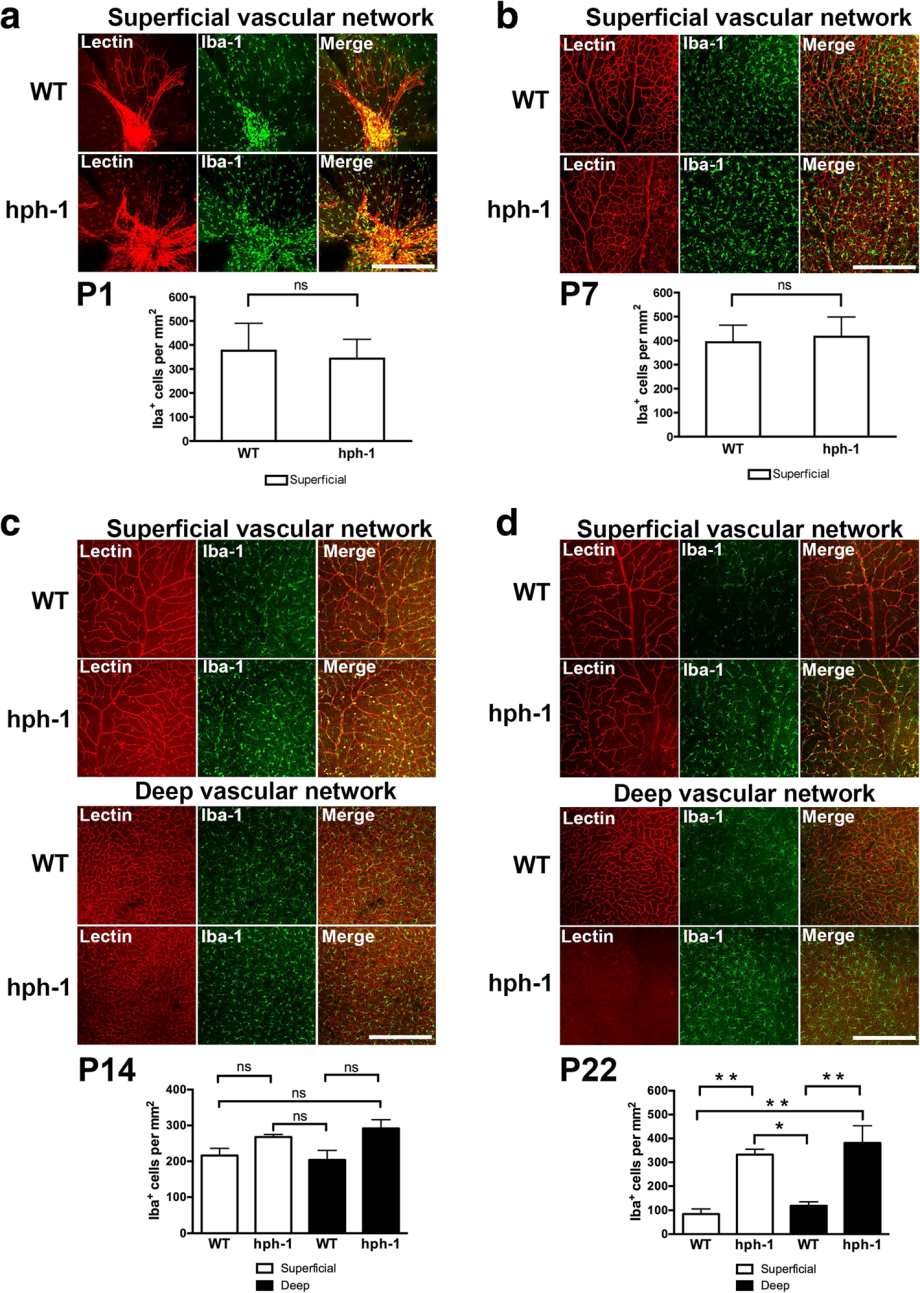
Microvascular degeneration in the retina of the tetrahydrobiopterin (BH4) deficient mice is associated with an augmentation on activated microglial cells with age. Representative confocal images from retinas showing immunoreactivity to TRITC-conjugated lectin endothelial cell marker *Bandeiraea simplicifolia* (red) and microglial marker Iba-1 (green) in the superficial (**a**–**d**) and deep (**c**, **d**) retinal vascular complexes from controls (WT) and *hph-1* mice at different postnatal ages (P1, P7, P14, and P22). Merged images show co-localization in yellow. **d** Iba-1 immunopositive cells were significantly increased in the superficial and deep retinal vascular complexes and associated with a dramatic absence of the deep microvasculature in *hph-1* mice at 22-days-old. Histogram (**a**–**d**) compiles average number of Iba-1-positive cells per mm^2^ quantified in three different fields on the superficial and/or deep vascular complexes for each group (*n* = 4–5 per group). ns not significant, *p < 0.05, **p < 0.01 vs control. Scale bar = 100 μm

Since a deficiency in BH4 can increase ROS production [6], which results in inflammatory disorders followed by endothelial dysfunction and tissue injury [8], we decided to evaluate the number of inflammatory cells in retinal flatmounts from *hph-1* and WT stained with Iba-1, a specific marker for microglial cells. The number of retinal microglia in *hph-1* mice at P1, P7, and P14 was very similar in comparison to the WT mice at the same ages (Fig. 3a–c). However, the number of microglial cells in retinas from *hph-1* animals at P22 was significantly increased (*p* < 0.01) when compared with the same age control mice (Fig. 3d). Interestingly, these findings suggest a strong correlation between the number of inflammatory cells and retinal microvascular degeneration.

### Pro-inflammatory factors are increased meanwhile protective endothelial cells factors decrease in the retina of *hph-1* mice

**Fig. 4:**
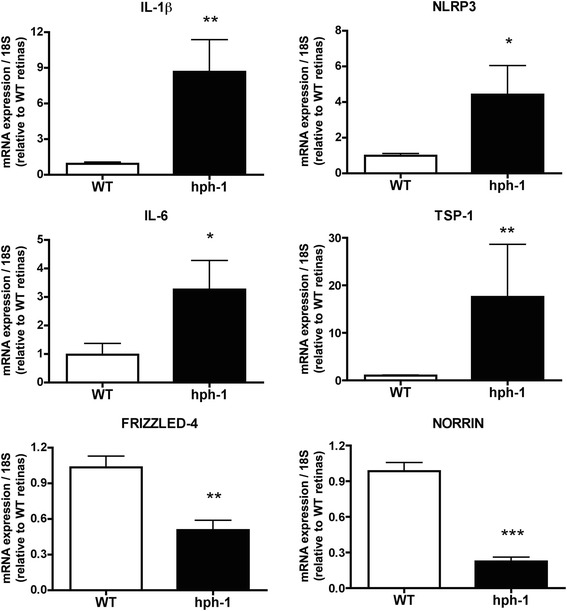
Tetrahydrobiopterin (BH4) deficiency is associated with upregulated expression of pro-inflammatory mediators in the retina of *hph-1* mice. Quantitative real-time PCR analysis was performed on whole retinas at P22 from control (WT) and *hph-1* animals; control values were set at 1. A significant increase in retinal mRNA expression of IL-1β (*p* < 0.005), NLRP3 (*p* < 0.03), IL-6 (*p* < 0.04), and TSP-1 (*p* < 0.005) and decrease on Norrin (*p* < 0.0001), and its receptor Frizzled 4 (FZD4; *p* < 0.001) was detected in *hph-1* retinas compared with the control. Values are mean ± SEM of *n* = 9–10 animals per group. The fold changes were normalized to 18S as internal control. Significant differences (*p* value) in the mRNA levels between control and *hph-1* mice are shown in the graphs; *p < 0.05, **p < 0.001, ***p < 0.0001 compared to the control

The next step was to determine whether activated inflammatory cells are associated with an overproduction of pro-inflammatory factors that may be involved in endothelial cell injury as we and others previously demonstrated [11, 38, 39]. Therefore, we evaluated the production of some pro-inflammatory cytokines as well as another’s possible factors involved in the vasculature damage in the retinas from *hph-1* mice at P7, P14, and P22 (Fig. 4 and Additional file 5: Figure S5). qPCR analysis revealed a significant increase in the mRNA expression of the pro-inflammatory cytokine IL-1β (2.91-fold; *p* < 0.01; *n* = 9 and 8.6-fold; *p* < 0.005; *n* = 10), and the major inflammasome protein NOD-like receptor family pyrin domain containing-3 (NLRP3; 1.67-fold; *p* < 0.03; *n* = 9 and 4.4-fold; *p* < 0.03; *n* = 9) in retinas from *hph-1* mice at P14 and P22, respectively, compared to the WT (Fig. 4 and Additional file 5: Figure S5). The expression of the pro-inflammatory cytokine IL-6 was particularly augmented at P22 (3.2-fold; *p* < 0.04; *n* = 10), but not at P14 (*p* = 0.097; *n* = 7). No significant changes in the expression levels of IL-1β, NLRP3 and IL-6 respect to the control were detected in retinal samples at P7 (Additional file 5: Figure S5). Interestingly, Norrin and its receptor Frizzled-4 (FZD4) strongly involved in the control of retinal vascular development and survival [40, 41] were decreased by 0.36-fold (*p* < 0.01; *n* = 10) and 0.2-fold (*p* < 0.0001; *n* = 12) and 0.55-fold (*p* < 0.01; *n* = 10) and 0.51-fold (*p* < 0.001; *n* = 6) respectively in retinas from *hph-1* mice at P14 and P22 (Fig. 4 and Additional file 5: Figure S5). Norrin showed an increased expression by 2.28-fold (*p* < 0.013; *n* = 10) in retinal samples of *hph-1* mice at P7, however, no significant changes in the expression of FZD4 were detected at this same age (*p* = 0.62; *n* = 10; Additional file 5: Figure S5). Furthermore, thrombospondin-1 (TSP-1), a matricellular glycoprotein secreted in response to injury and stress in the retina with anti-angiogenic properties and involved in vascular injury [15, 20, 21] was significantly augmented by 2.09-fold (*p* < 0.004; *n* = 10) and 17.5-fold (*p* < 0.005; *n* = 10) in the retinas from *hph-1* mice at P14 and P22, respectively when compared to control animals (Fig. 4 and Additional file 5: Figure S5).

### Activated microglial cells produces factors associated to microvascular degeneration in the retina of *hph-1* mice

**Fig. 5:**
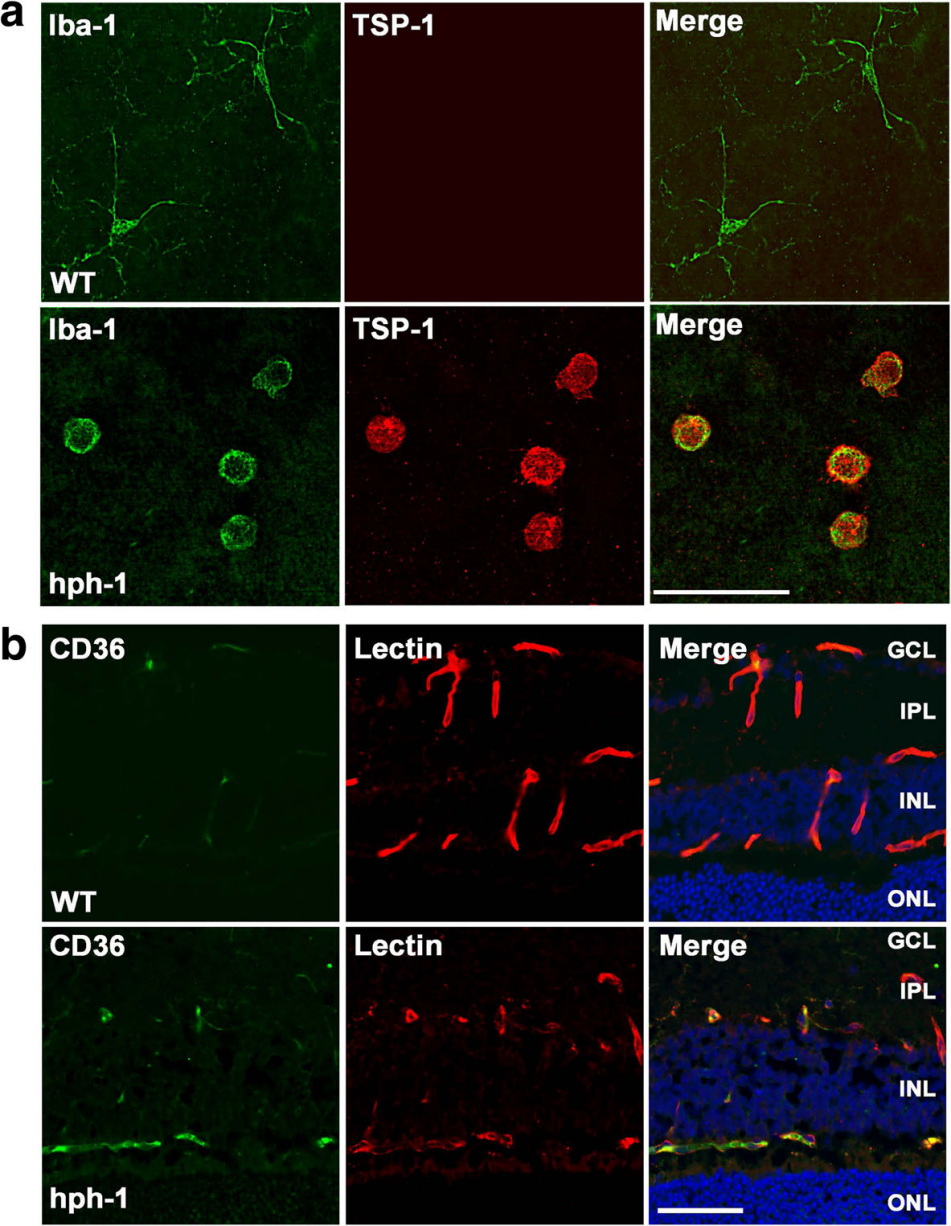
Thrombospondin-1 (TSP-1) is localized in microglial cells and its receptor CD36 in the retinal microvasculature from BH4 deficient mice. **a** Representative confocal images from retinal flatmounts showing immunoreactivity of TSP-1 (red) and Iba-1 (green). Co-staining of TSP-1 with Iba-1 was detected mainly on activated microglial cells (yellow) localized in the nerve fiber layer (NFL) or the ganglion cell layer (GCL) in retinas from *hph-1* mice but not in the control (WT). Scale bar = 50 μm. **b** Representative confocal images of CD36 receptor (green) and lectin (red) merged with DAPI (blue and yellow) in retinal cryosections from control and *hph-1* mice at 22-day-old (*n* = 3 per group). CD36 receptor staining was highly detected in the damaged microvasculature present in *hph-1* mice (yellow), but not in the WT mice. Scale bar = 50 μm

The increased number of microglial cells and inflammatory factors is suggestive of an acute inflammatory response in the retinas of *hph-1* mice (Figs. 3 and 4). We focus our attention particularly on IL-1β and TSP-1, based in the fact that both molecules have been previously implicated in retinal microvascular degeneration [11, 15, 20]. Double immunolabeling in the retina by using different specific markers showed that both IL-1β and TSP-1 proteins were positively labeled in microglial cells (Iba-1 immunopositives) from *hph-1* mice (Fig. 5a and Additional file 6: Figure S6A); while the immunoreactivity of both proteins in microglia cells in the retinas from WT mice was very weak (Fig. 5a and Additional file 6: Figure S6A). Because it has been proposed that IL-1β derived from activated microglia induces microvascular injury indirectly through the release of pro-apoptotic/repulsive factor semaphorin-3A (Sema3A) in the retina [11], we decide to evaluate the expression of Sema3A in retinas from *hph-1* mice at P22. Surprisingly, mRNA expression of Sema3A was significantly decreased (0.5-fold; *p* < 0.0001; *n* = 10) suggesting that Sema3A is probably not involved in the vascular injury process in the retina from *hph-1* mice (Additional file 6: Figure S6B).

### TSP-1 overexpression is associated with microvasculature degeneration and suppressed with BH4 supplementation

**Fig. 6:**
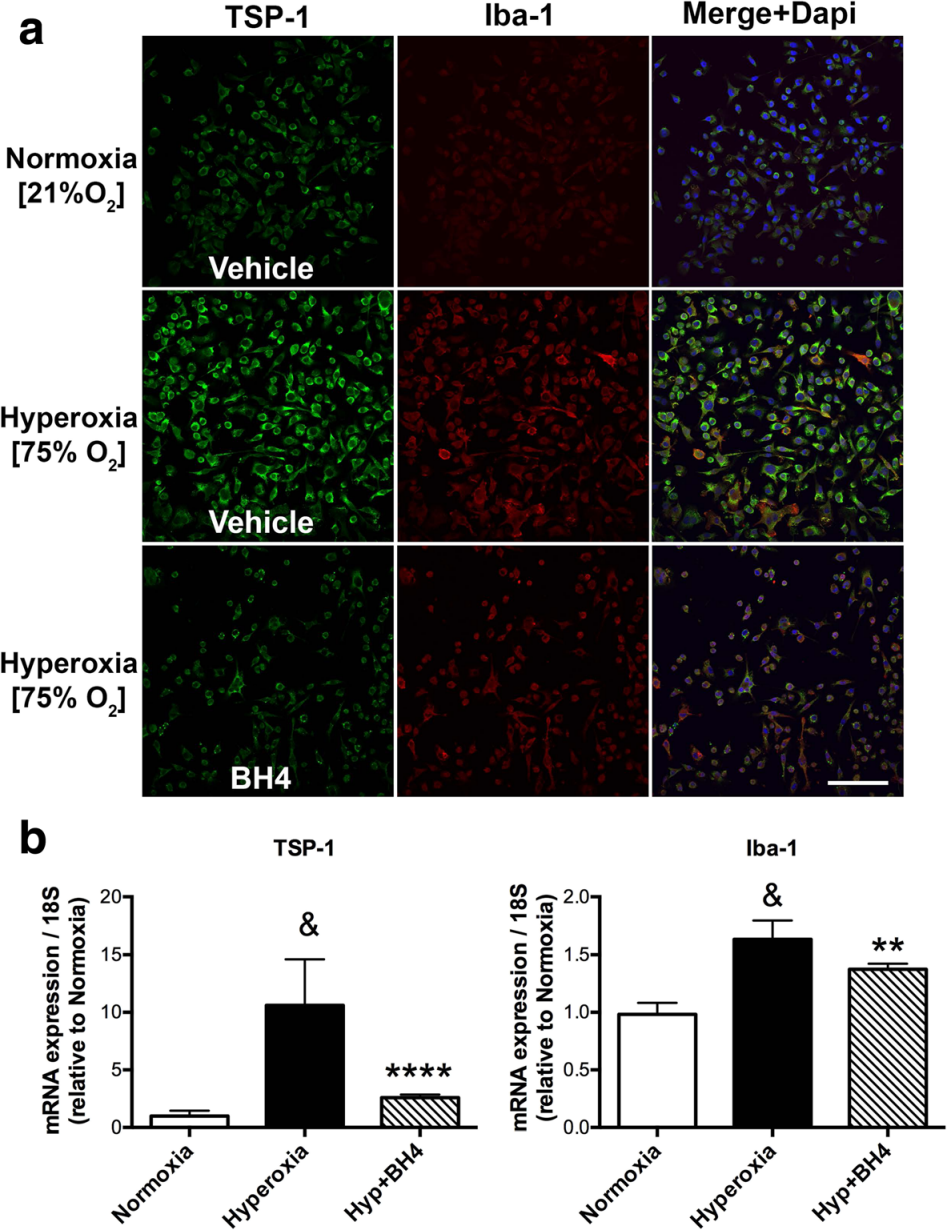
TSP-1 is released from microglial cells exposed to hyperoxia in vitro, but suppressed by BH4 supplementation. **a** Representative confocal images showing immunoreactivity of TSP-1 (green) and Iba-1 (red) merged with DAPI (blue and yellow) in microglial cell (SIM-A9) cultures exposed to normoxia (21% O_2_) or hyperoxia (75% O_2_) and supplemented with vehicle (PBS 1×, sterile) or with BH4 (100 μM) during 24 h. Scale bar = 100 μm. Protein staining (**a**) and mRNA expression (**b**) evaluated by qPCR in TSP-1 and Iba-1 were significantly decreased in microglial cells exposed to hyperoxia and supplemented with BH4. Values in the graphs represent the mean ± SEM of *n* = 6–8 experiments per group. The fold changes were normalized to 18S as internal control. Significant differences (*p* value) in the mRNA levels between normoxia and hyperoxia are shown in the graphs; ^&^ *p* < 0.0001 compared to the control, **p < 0.001 and ****p < 0.0001 vs hyperoxia

**Fig. 7:**
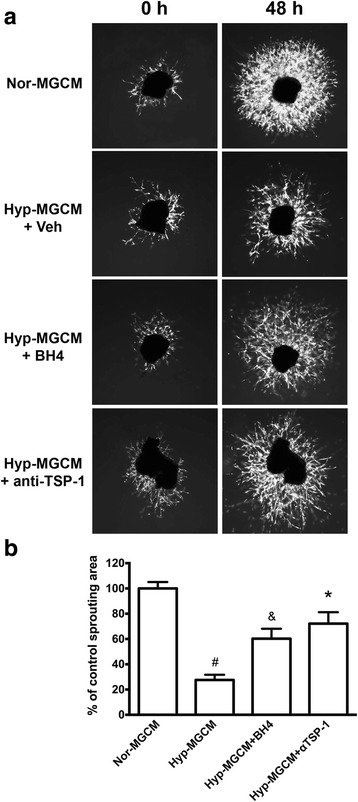
Antiangiogenic effect of hyperoxic microglia-conditioned media containing TSP-1 is abolished by the immunoneutralization of TSP-1 or with pre-incubation of microglial cells with BH4. **a** Representative micrographs of choroidal explants stimulated with conditioned media (CM) from microglial cells (MG) previously exposed to normoxia (Nor-MGCM) or hyperoxia stimulated with vehicle (Hyp-MGCM + Veh) or BH4 (Hyp-MGCM + BH4) and/or in presence of a TSP-1 neutralizing monoclonal antibody (1.2 μg/ml, Hyp-MGCM + anti-TSP-1) for 48 h. Choroidal explants cultured in matrigel containing growth factors and treated with Nor-MGCM displayed increased sprouting, whereas explants exposed to Hyp-MGCM + Veh exhibited a significant diminished sprouting growth. The choroidal explants incubated with Hyp-MGCM + BH4 or in presence of a TSP-1 neutralizing antibody showed a significant augmentation in the sprouting compared to Hyp-MGCM + Veh. **b** The histogram depicts the quantification of choroidal explant sprouting (^#^ *p* < 0.0001 compared to Nor-MGCM, ^&^ *p* < 0.05, and *p < 0.001 compared to Hyp-MGCM + Veh; *n* = 5 per group)

Therefore, we focus our attention on TSP-1, a well-known anti-angiogenic factor that can directly act on endothelial cells by inhibiting their proliferation and migration or promoting their apoptosis [42–44]. Previous studies in our laboratory have already demonstrated that nitrative stress can induce TSP-1 upregulation which cause retinal microvascular degeneration in a model of oxygen-ischemic retinopathy [15]. Here, TSP-1 was highly immuno localized in activated microglial cells in the retinas from *hph-1* mice compared to WT (Fig. 5a). Besides this, the TSP-1 receptor (CD36) was strongly localized in the retinal microvasculature damaged in BH4-deficient mice (Fig. 5b), suggesting that TSP-1 secreted by activated microglial cells could act through its receptor highly expressed in endothelium, and be part of the mechanism implicated in the detrimental effect of the microvasculature. To evaluate this hypothesis, microglial cell cultures were exposed to hyperoxia-induced oxidative stress to induce its activation [11] and decrease BH4 levels as recently demonstrated [45]. Microglial cell cultures under hyperoxia were supplemented or not with an effective dose of BH4 [27] (100 μM; Fig. 6a). After 24 h of incubation, microglial cells lysates were used to evaluate TSP-1 expression by qPCR (Fig. 6b), while condition media was used on choroidal explants to evaluate the angiostatic activity in presence or absence of a neutralizing TSP-1 antibody (Fig. 7). As we expected, exposure of microglial cells to hyperoxia-induced oxidative stress for 24 h revealed a robust increase in TSP-1 mRNA expression (in lysates cells) and protein (by immunohistochemistry) (Fig. 6) compared to normoxia (21% O_2_).

Interestingly, conditioned media (which contains TSP-1) from hyperoxia-exposed microglia was directly cytotoxic to the microvasculature from choroidal explants or microvascular endothelial cells in culture (Fig. 7). Conversely, BH4 supplementation markedly prevented hyperoxia-induced microglial activation as attested by diminished Iba-1 and TSP-1 expression (Fig. 6b) and prevented microvascular injury in choroidal explants (Fig. 7; corroborated by using a specific neutralizing TSP-1 antibody). Finally, by using the oxygen-induced retinopathy (OIR) model characterized by retinal vaso-obliteration associated with microglial activation during the first phase of the pathology [46]. We demonstrated the intravitreal injection of BH4 (100 μM final concentration) significantly decrease (*p* < 0.0002) retinal vascular degeneration after 48 h of hyperoxia (Additional file 7: Figure S7).

All these results show that in the absence of BH4, an augmented secretion of the anti-angiogenic TSP-1 derived from activated microglia (Fig. 5) contributes significantly to microvascular degeneration in the retina of *hph-1* mice.

## Discussion

Tetrahydrobiopterin (BH4) is an essential cofactor present in all tissues in the body and plays a key role in a number of biological processes including endothelial cell survival and immune response [47–49]. In this study, BH4-deficient mice showed a series of morphological and functional changes in the eye including a decremented reduction in the globe size, hypertrophy iris, persistence of fetal hyaloid vasculature and microvascular degeneration. We focus our study particularly on retinal microvascular injury based in the fact that BH4 deficiency has been associated with an augmented inflammatory response followed by endothelial dysfunction and tissue injury observed in a variety of experimental and clinical vascular diseases [7, 8]. Similar to a previous report [45], we have not detected major differences in vascular development between wild-type and *hph-1* mice in both superficial and deep vascular plexuses until postnatal day 14.

Surprisingly, in retinas of BH4-deficient mice evaluated at P22, we detected a dramatic microvascular degeneration in the superficial and deep vascular plexuses associated with an exacerbated inflammatory response characterized by an augmented number of microglial cells, as well as, a high production of pro-inflammatory factors. Therefore, we propose that in the absence of BH4, microglial cells could become activated and secrete factors that play a key role in the microvascular degeneration in the eye of these animals. Microglial cells have been reported to contribute significantly in retinal vascular development [50, 51]; however, their participation in microvascular degeneration has also been demonstrated [11, 39].

Under severe insults, microglia become overactivated and functions as a prominent source of cytotoxic oxidant stress and pro-inflammatory factors implicated in microvascular alterations in several ocular diseases in humans [52] and animal models [14, 53, 54]. In this regard, high levels of IL-6, NLRP3, IL-1β, and TSP-1 detected in the retinas from *hph-1* mice were considered as possible candidates involved in microvascular degeneration. We focus our attention on IL-1β and TSP-1, based on the fact that both molecules are largely implicated in retinal microvascular degeneration [11, 15, 20]. For instance, a previous study in our laboratory showed that overactivated microglial cells become the main source of IL-1β, which is implicated in retinal microvascular degeneration not directly, but through the release of pro-apoptotic/repulsive factor Semaphorin 3A (Sema3A) from adjacent neurons [11]. Surprisingly, in the present study, the levels of Sema3A were found to be decrease, suggesting that IL-1β/Sema3A signaling is probably not implicated in microvascular degeneration in *hph-1* mice.

On the other hand, TSP-1, a matricelllular glycoprotein has also proven to have a potent anti-angiogenic activity in the eye. For intance, mice deficient in TSP-1 have shown an increased retinal vascular density [20], whereas overexpression of TSP-1 in the eye results in the attenuation of the retinal vascular development [55]. Previous reports have shown that under oxidative stress, TSP-1 is secreted and responsible to induce retinal microvascular degeneration [15, 55] by acting through its receptor CD36, localized on endothelium [56, 57]. In this study, we have detected augmented levels of TSP-1 on activated microglial cells, as well as, its CD36 receptor localized on injured retinal microvasculature from BH4-deficient mice. Per these findings, we suggested that in BH-deficient mice, retinal microglia activated is an important source of TSP-1, which is directly responsible to induce retinal microvascular degeneration in the superficial and deep vascular plexuses.

To demonstrate this hypothesis, microglial cells were in vitro cultured under hyperoxic stress conditions to suppress BH4 levels as previously reported [45]. We showed that in response to hyperoxic stress, microglial cells become activated and secreted high levels of TSP-1, which in turn caused choroidal microvascular degeneration on the ex-vivo choroidal sprouting assay. This angiostatic effect of microglia-derived TSP-1 was suppressed by BH4 supplementation and/or by using a specific neutralizing TSP-1 antibody. Importantly, these findings were consistent with previous studies demonstrating that BH4 is critical to preserve the function and regenerative capacity on endothelial progenitor cells, at least in part, by suppressing oxidative stress and TSP-1 [17]. Additional to this, we also have shown that BH4 supplementation decreased microglial activation and TSP-1 release in vitro and significantly diminished oxygen-induced microvascular injury in vivo. This protective effect of BH4 on the retinal vasculature in vivo could be explained in part due to a possible inhibition in the expression of TSP-1, which has been shown to be augmented during the vascular degeneration period that occurs approximately after 24 h of hyperoxia in the oxygen-induced retinopathy (OIR) model [15]. Likewise, TSP-1-deficient mice are less susceptible to oxygen-induced degeneration [20].

Besides this, our findings do not fully exclude the contribution of other factors implicated on microvascular damage. For instance, retinal alterations in absence of BH4 were associated with a decreased expression of protective factors such as Wnt ligand Norrin and its receptor FZD4 that play an important role in the maintenance of the deep retinal vasculature. Norrin is a small protein secreted exclusively in Müller cells and is critical in the formation of the deep capillary network during retinal development [40]. Norrin exerts its angiogenic effects by binding to the Wnt receptor FZD4. That, in cooperation with its co-receptors Lrp5 and the TSPAN12, activates the canonical Wnt/β-catenin signaling pathway in Müller cells or microvascular endothelial cells and triggers the expression of targets genes involved in vascular growth, remodeling, and maintenance [40, 58]. Accordingly, a deficiency in Norrin, FZD4, Lrp5/6, or TSPAN12 leads to a marked deficiency in intraretinal vascular development [40]. Moreover, canonical Wnt signaling is also implicated in regression of embryonic hyaloid vessels in the eye [59]. Mice deficient in Wnt ligand norrin or Wnt co-receptor Lrp5 have persistent embryonic hyaloid vessels in the eye [60, 61]. These findings can in part explain the association between low levels of norrin and the presence of hyaloid vessels in young adult *hph-1* mice at P22. Therefore, based on all these previous facts, we propose that in addition to TSP-1, the decreased expression of Norrin and FZD4 might be part of the mechanism implicated in the detrimental effect of the deep microvasculature and the persistence of hyaloid vessels in the retina from *hph-1* mice, but this needs to be further investigated.

## Conclusion

In summary, our findings indicate that BH4 is a key cofactor regulator of inflammatory and anti-angiogenic factors that play an important function in the maintenance of retinal microvasculature. In the absence of BH4, an augmented secretion of activated microglia-derived TSP-1 is responsible for the retinal microvascular alterations in *hph-1* mice. BH4 supplementation was able to suppress the excessive antiangiogenic activity of TSP-1 and modulate the inflammatory activity of microglial cells under hyperoxic conditions.

## Additional files

Additional file 1: Figure S1. Morphology of the eyes from *hph-1* and WT mice. (A) Representative images of the eyeballs from wild type (WT) and *hph-1* mice analyzed by H&E staining at postnatal day 1, 7, and 14. The pictures show a reduction in the size, as well as, appearance of hypertrophy in the iris and persistence of fetal hyaloid vasculature in the eyes of *hph-1* mice. (PDF 297 kb)

Additional file 2: Figure S2. Tetrahydrobiopterin (BH4) content in *hph-1* mice at early ages. BH4 content (ng/mg of tissue) in retinas from control (WT) and *hph-1* mice at postnatal day 7 and 14 (P7 and P14) was measured by using liquid chromatography tandem mass spectrometry (LC-MS/MS). The levels of BH4 were significantly decreased by ~ 90% in the retinas of *hph-1* at P7 and P14 compared with WT mice. Results showed in the histograms are expressed as means ± SEM of *n* = 5 mice for each group. **p < 0.005 and ***p < 0.0001 compared to control. (PDF 109 kb)

Additional file 3: Figure S3. Retinal flat-mounts from *hph-1* and WT mice at different ages. Representative images of whole-mounted retinas labeled with TRITC-conjugated lectin endothelial cell marker *Bandeiraea simplicifolia* showing retinal vasculature from controls (WT) and *hph-1* mice at different postnatal ages (P1, P7, P14, and P22). Note that at P1, the vessels in the retina are mainly represented by the hyaloid vasculature. (PDF 263 kb)

Additional file 4: Figure S4. Vascular density in *hph-1* and WT mice at early ages. The quantification of the vascular density in both superficial and deep retinal vascular complexes was not significant in *hph-1* mice compared to the WT control at P1, P7, and P14. Results showed in the histograms are expressed as means ± SEM of *n* = 3–4 retinas for each group. ns = not significant compared to the control. (PDF 193 kb)

Additional file 5: Figure S5. Tetrahydrobiopterin (BH4) deficiency is associated with upregulated expression of pro-inflammatory mediators at P14. Quantitative real-time PCR analysis was performed on whole retinas at P7 and P14 from control (WT) and *hph-1* animals; control values were set at 1. A significant increase in retinal mRNA expression of IL-1β (*p* < 0.01), NLRP3 (*p* < 0.03), and TSP-1 (*p* < 0.005), but not on IL-6 (*p* < 0.04) was detected in *hph-1* mice at P14. Decrease on Norrin (*p* < 0.01) and its receptor Frizzled 4 (FZD4; *p* < 0.001) were detected in *hph-1* retinas compared with the control at P14. Norrin was significantly augmented in hph-1 mice at P7. Values are mean ± SEM of *n* = 9–10 animals per group. The fold changes were normalized to 18S as internal control. Significant differences (*p* value) in the mRNA levels between control and *hph-1* mice are shown in the graphs; *p < 0.05 and **p < 0.001 compared to the control. (PDF 252 kb)

Additional file 6: Figure S6. Interleukin-1β (IL-1β) is localized on microglial cells and Semaphorin 3A (Sema3A) is decreased in the retinas from BH4 deficient mice. (A) Representative confocal images showing immunoreactivity for IL-1β (green) and Iba-1 (red) merged with DAPI (blue and yellow) in retinal cryosections from control (WT) and *hph-1* mice at 22-day-old (*n* = 3 per group). Co-staining of IL-1β with Iba-1 was detected on microglial cells localized in the ganglion cell layer (GCL) and deep plexiform layer (IPL) in retinas from *hph-1* mice but not in WT mice. Scale bar = 50 μm. (B) Quantitative real-time PCR analysis was performed on whole retinas at P22 from control (WT) and *hph-1* animals; control values were set at 1. A significant decrease in retinal mRNA expression of Sema3A (***p < 0.0001; *n* = 10), was detected in *hph-1* retinas compared with the control. Values are mean ± SEM. The fold changes were normalized to 18S as internal control. (PDF 333 kb)

Additional file 7: Figure S7. Intraocular supplementation of tetrahydrobiopterin (BH4) prevents retinal vasoobliteration in mice exposed to oxygen-induced retinopathy (OIR). Representative images of flat-mounted retinas labeled with fluorescein-labeled *Griffonia Simplicifolia* Lectin 1 (GSL 1), isolectin B4 to examine vasoobliteration in animals exposed to 75% oxygen from P7 until P9. Animals were intravitreally injected at P7 with 100 μM of BH4 or vehicle (PBS steril 1×) and retinas analyzed at P9. Significant differences (*p* value) in the vasoobliterated area between vehicle and BH4 treatment after 48 h of hyperoxia are shown in the graphs; ***p < 0.0002 compared to hyperoxia. (PDF 292 kb)

## Abbreviations

(6R)-BH4: (6R)-5,6,7,8-Tetrahydrobiopterin dihydrochloride
BH4: Tetrahydrobiopterin
DVC: Deep vascular complex
ENOS: Nitric oxide synthase
FZD4: Frizzled-4
GTP: Guanosine triphosphate
GTPCH I: GTP cyclohydrolase I
IL-1β: Interleukin-1β
LC-MS/MS: Liquid chromatography tandem mass spectrometry
NO: Nitric oxide
OIR: Oxygen-induced retinopathy
PBS: Phosphate-buffered saline
ROS: Reactive oxygen species
Sema3A: Semaphorin-3A
SIM-A9: Microglia cell line
SVC: Superficial vascular complex
TSP-1: Thrombospondin-1
