# Supplementary figures and images for: Tetrahydrobiopterin (BH4) deficiency is associated with augmented inflammation and microvascular degeneration in the retina
Source: J Neuroinflammation. 2017 Sep 6;14:181. doi: 10.1186/s12974-017-0955-x (PMC5586016; doi:10.1186/s12974-017-0955-x)

**Wild-Type**

**hph-1**

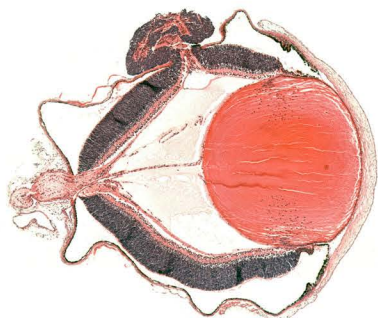

**P1**

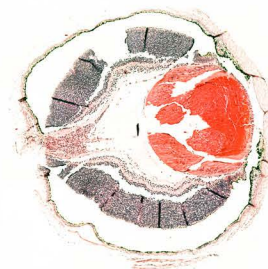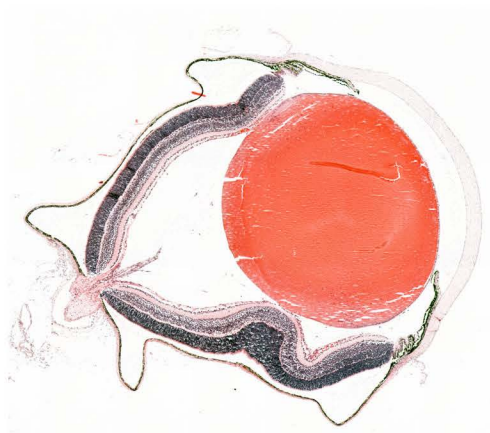

**P7**

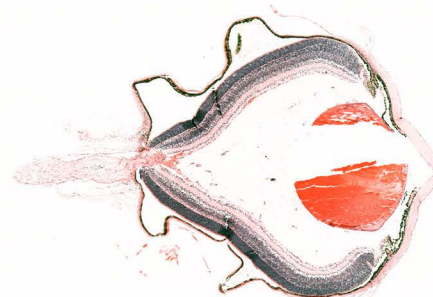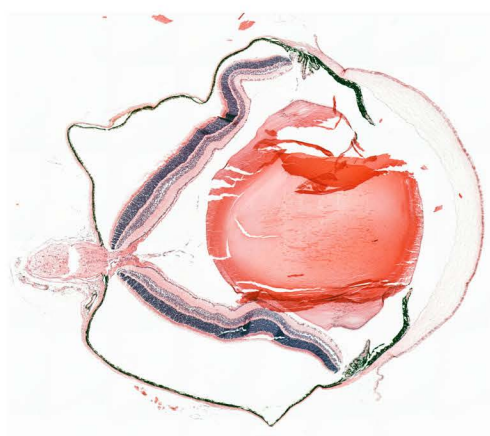

**P14**

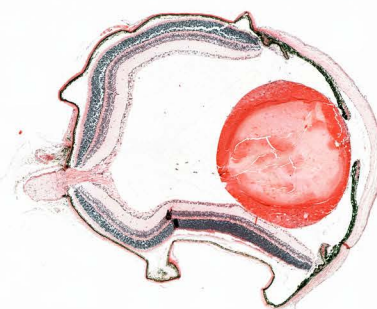

Supplement: Supplementary file 1 — Morphology of the eyes from hph-1 and WT mice. (A) Representative images of the eyeballs from wild type (WT) and hph-1 mice analyzed by H&E staining at postnatal day 1, 7, and 14. The pictures show a reduction in the size, as well as, appearance of hypertrophy in the iris and persistence of fetal hyaloid vasculature in the eyes of hph-1 mice. (PDF 297 kb) [file 12974_2017_955_MOESM1_ESM.pdf]

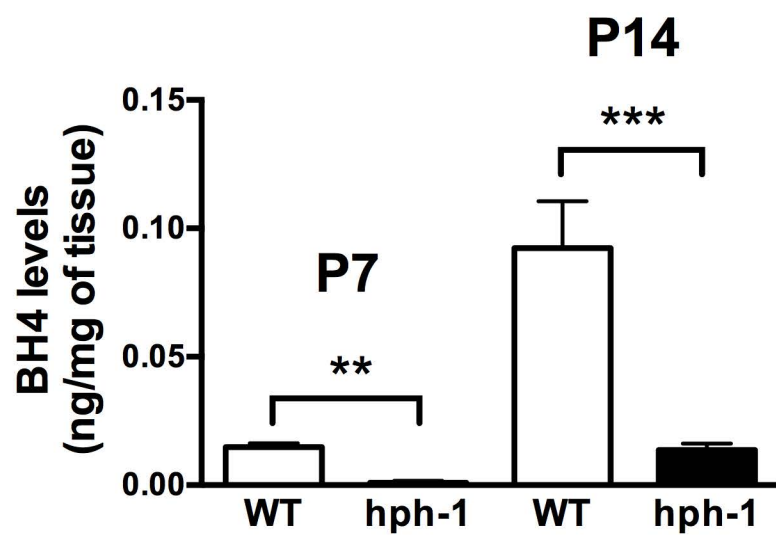

Supplement: Supplementary file 2 — Tetrahydrobiopterin (BH4) content in hph-1 mice at early ages. BH4 content (ng/mg of tissue) in retinas from control (WT) and hph-1 mice at postnatal day 7 and 14 (P7 and P14) was measured by using liquid chromatography tandem mass spectrometry (LC-MS/MS). The levels of BH4 were significantly decreased by ~ 90% in the retinas of hph-1 at P7 and P14 compared with WT mice. Results showed in the histograms are expressed as means ± SEM of n = 5 mice for each group. **p < 0.005 and ***p < 0.0001 compared to control. (PDF 109 kb) [file 12974_2017_955_MOESM2_ESM.pdf]

**P1**

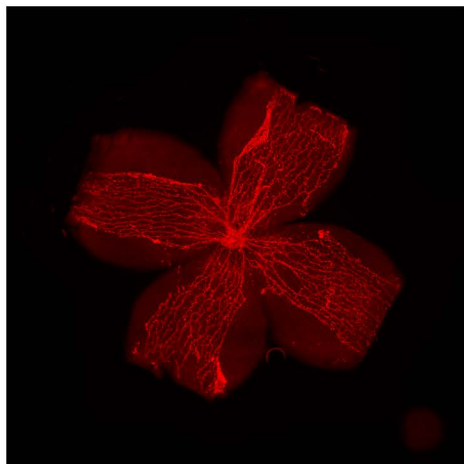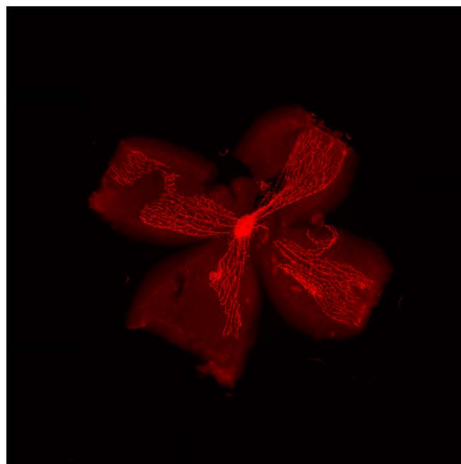

**P7**

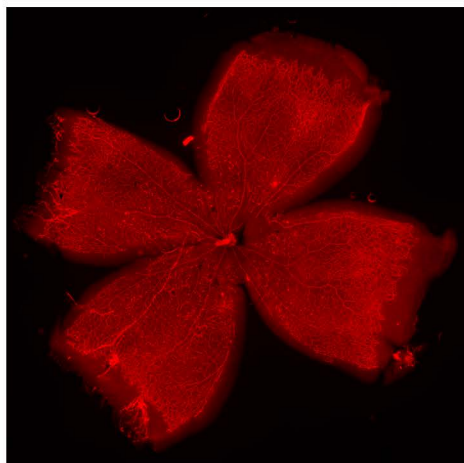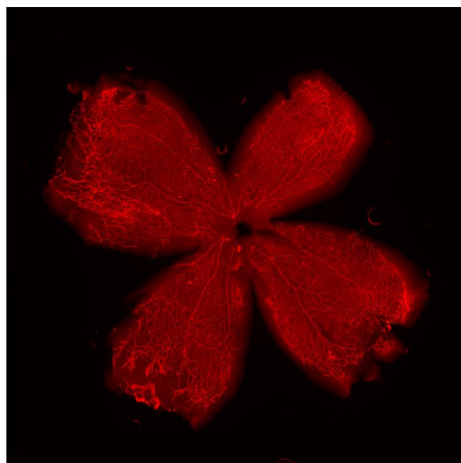

**P14**

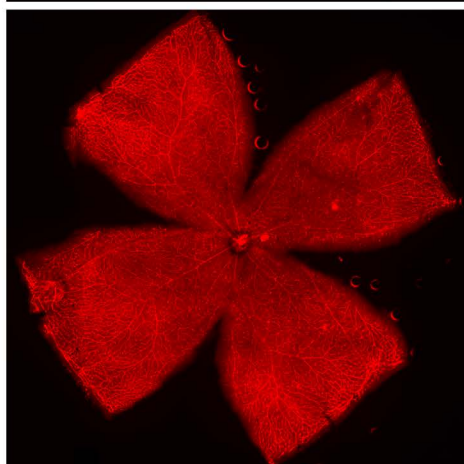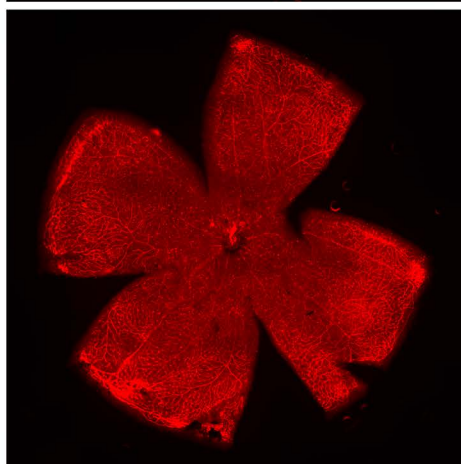

**P22**

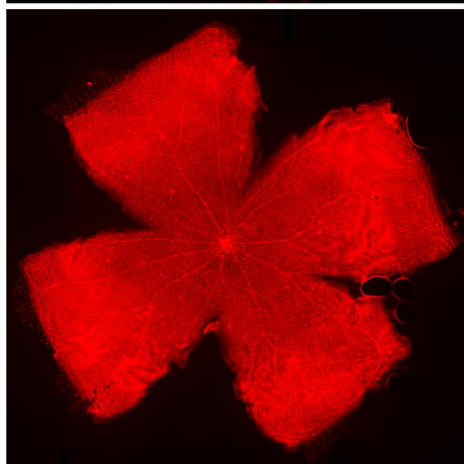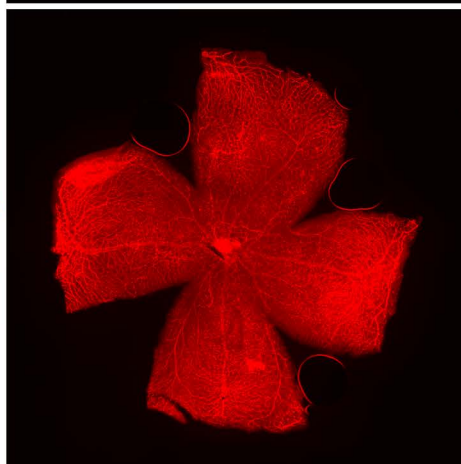

Supplement: Supplementary file 3 — Retinal flat-mounts from hph-1 and WT mice at different ages. Representative images of whole-mounted retinas labeled with TRITC-conjugated lectin endothelial cell marker Bandeiraea simplicifolia showing retinal vasculature from controls (WT) and hph-1 mice at different postnatal ages (P1, P7, P14, and P22). Note that at P1, the vessels in the retina are mainly represented by the hyaloid vasculature. (PDF 263 kb) [file 12974_2017_955_MOESM3_ESM.pdf]

**P1**

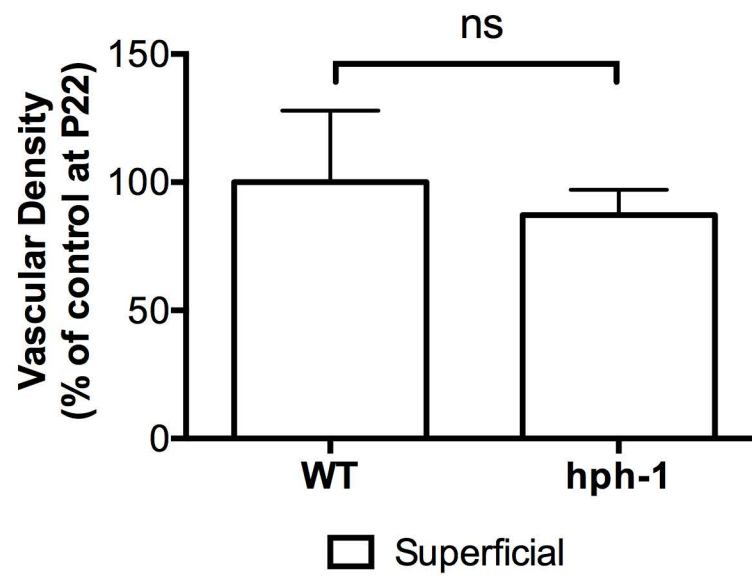

**P7**

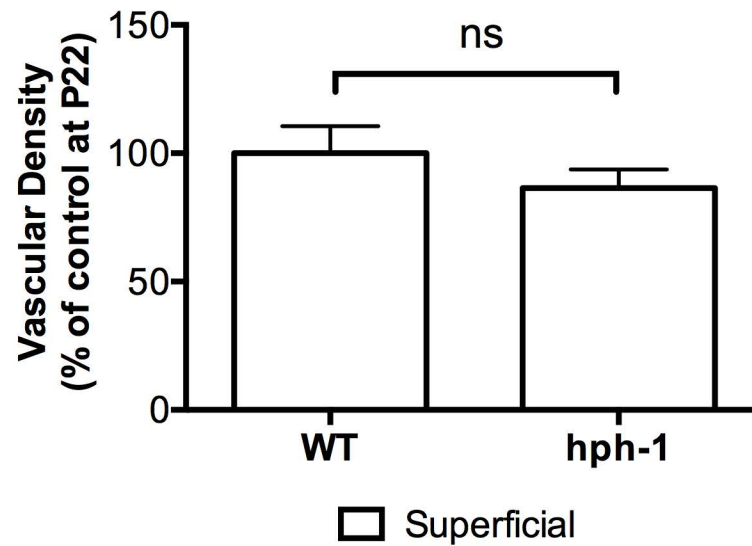

**P14**

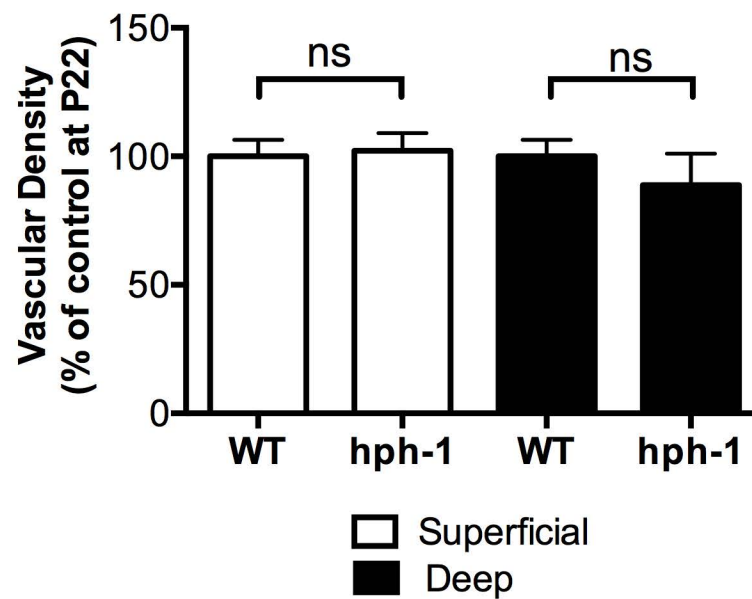

Supplement: Supplementary file 4 — Vascular density in hph-1 and WT mice at early ages. The quantification of the vascular density in both superficial and deep retinal vascular complexes was not significant in hph-1 mice compared to the WT control at P1, P7, and P14. Results showed in the histograms are expressed as means ± SEM of n = 3–4 retinas for each group. ns = not significant compared to the control. (PDF 193 kb) [file 12974_2017_955_MOESM4_ESM.pdf]

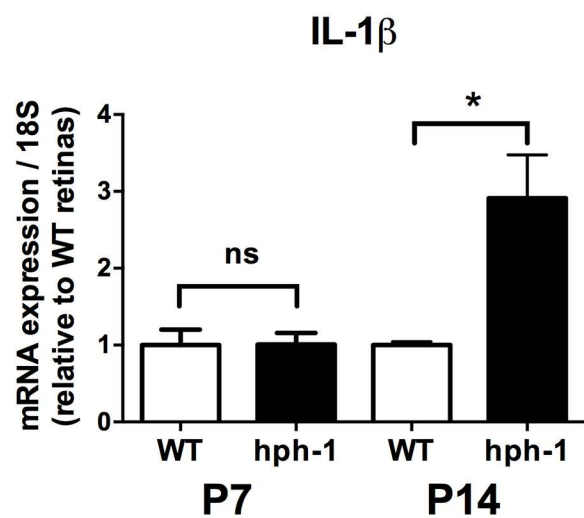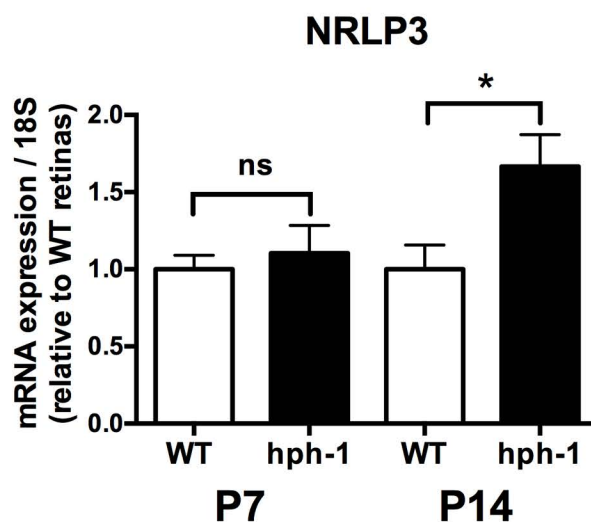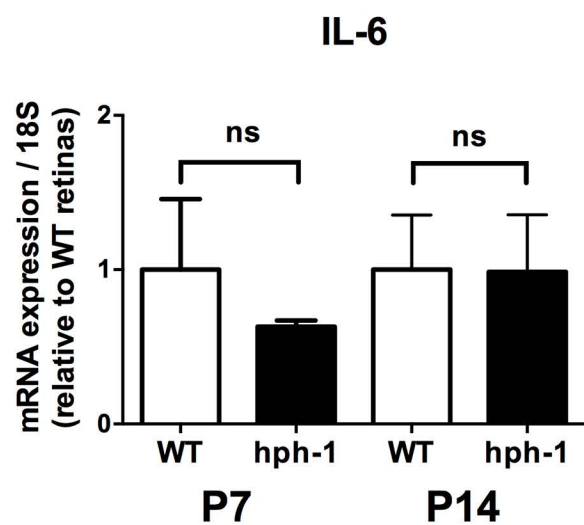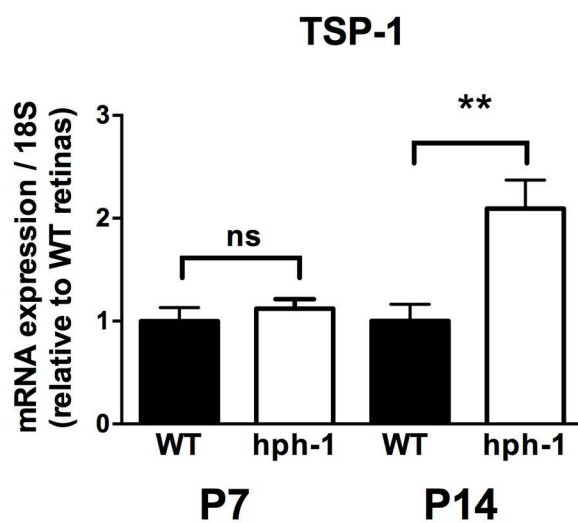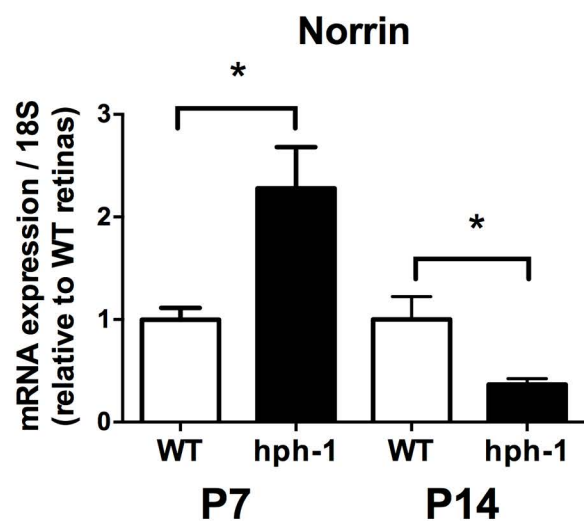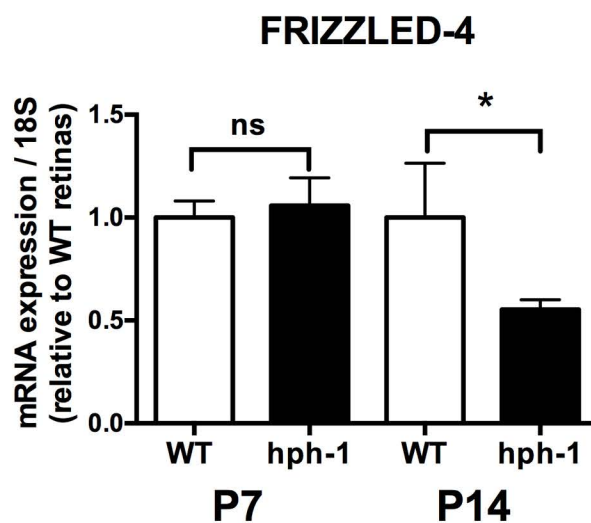

Supplementary Figure 5

Supplement: Supplementary file 5 — Tetrahydrobiopterin (BH4) deficiency is associated with upregulated expression of pro-inflammatory mediators at P14. Quantitative real-time PCR analysis was performed on whole retinas at P7 and P14 from control (WT) and hph-1 animals; control values were set at 1. A significant increase in retinal mRNA expression of IL-1β (p < 0.01), NLRP3 (p < 0.03), and TSP-1 (p < 0.005), but not on IL-6 (p < 0.04) was detected in hph-1 mice at P14. Decrease on Norrin (p < 0.01) and its receptor Frizzled 4 (FZD4; p < 0.001) were detected in hph-1 retinas compared with the control at P14. Norrin was significantly augmented in hph-1 mice at P7. Values are mean ± SEM of n = 9–10 animals per group. The fold changes were normalized to 18S as internal control. Significant differences (p value) in the mRNA levels between control and hph-1 mice are shown in the graphs; *p < 0.05 and **p < 0.001 compared to the control. (PDF 252 kb) [file 12974_2017_955_MOESM5_ESM.pdf]

**A**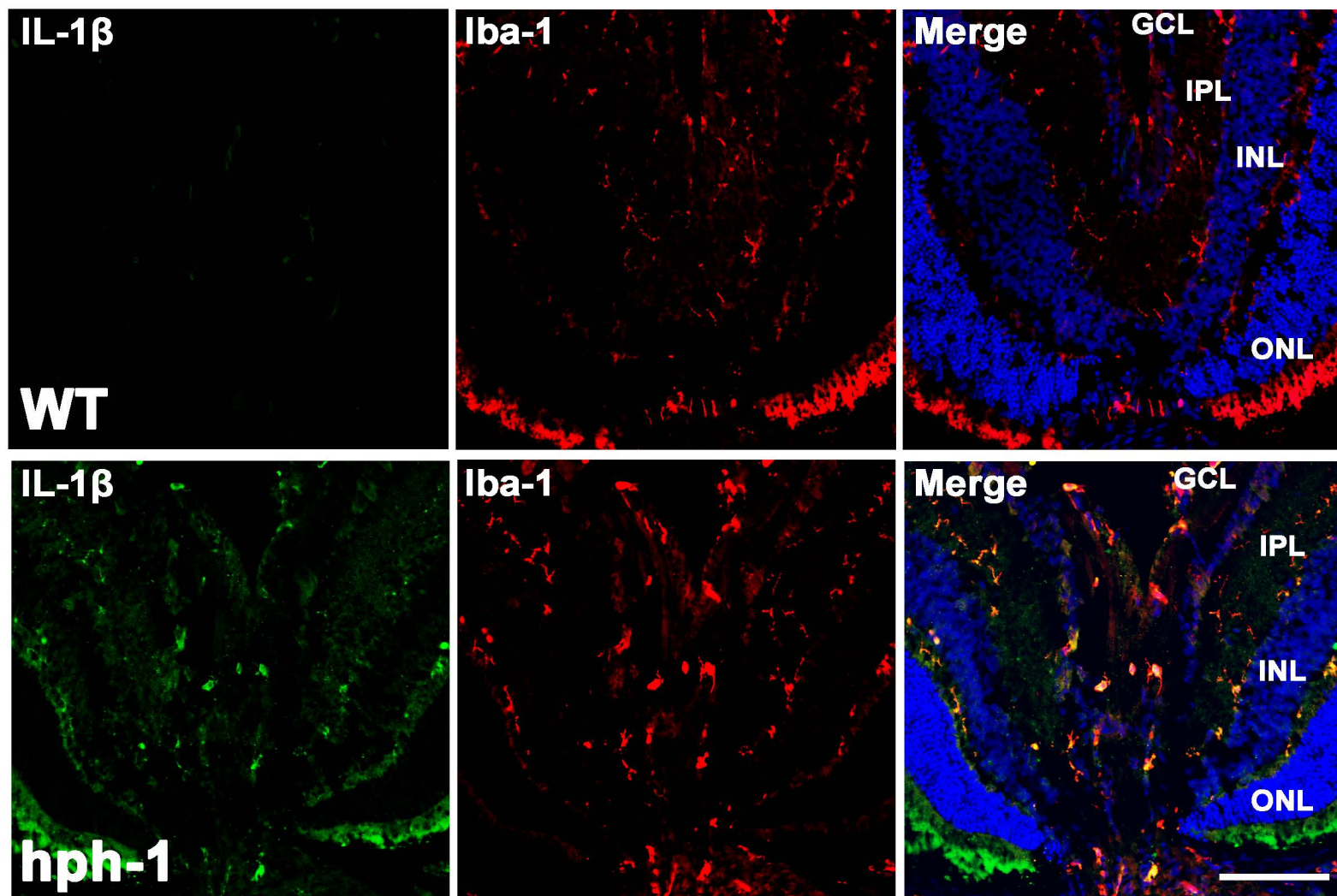**B**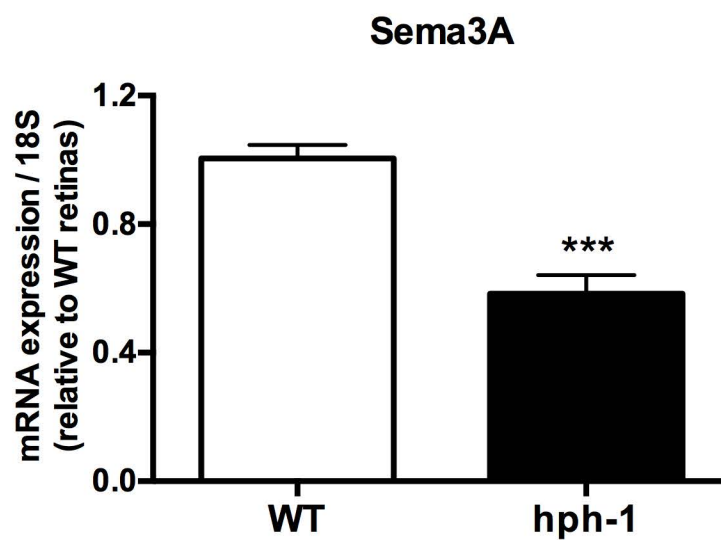

Supplement: Supplementary file 6 — Interleukin-1β (IL-1β) is localized on microglial cells and Semaphorin 3A (Sema3A) is decreased in the retinas from BH4 deficient mice. (A) Representative confocal images showing immunoreactivity for IL-1β (green) and Iba-1 (red) merged with DAPI (blue and yellow) in retinal cryosections from control (WT) and hph-1 mice at 22-day-old (n = 3 per group). Co-staining of IL-1β with Iba-1 was detected on microglial cells localized in the ganglion cell layer (GCL) and deep plexiform layer (IPL) in retinas from hph-1 mice but not in WT mice. Scale bar = 50 μm. (B) Quantitative real-time PCR analysis was performed on whole retinas at P22 from control (WT) and hph-1 animals; control values were set at 1. A significant decrease in retinal mRNA expression of Sema3A (***p < 0.0001; n = 10), was detected in hph-1 retinas compared with the control. Values are mean ± SEM. The fold changes were normalized to 18S as internal control. (PDF 333 kb) [file 12974_2017_955_MOESM6_ESM.pdf]

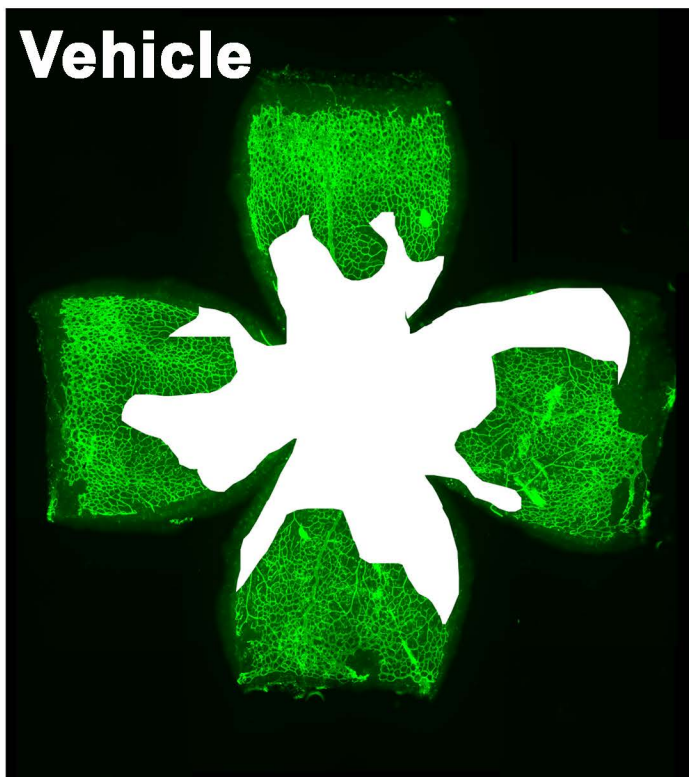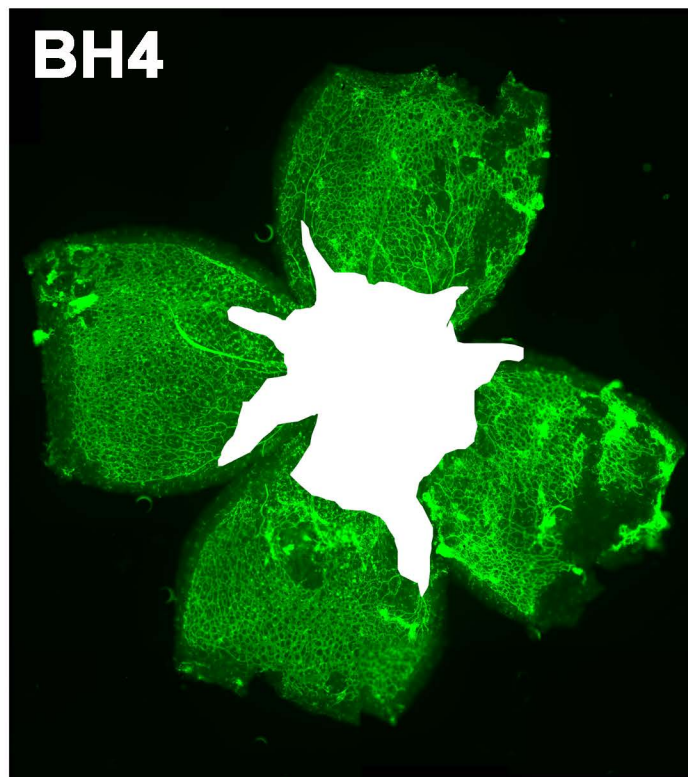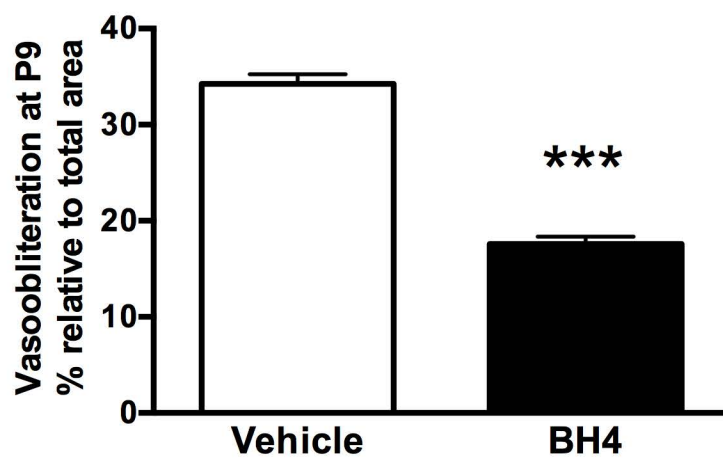

Supplement: Supplementary file 7 — Intraocular supplementation of tetrahydrobiopterin (BH4) prevents retinal vasoobliteration in mice exposed to oxygen-induced retinopathy (OIR). Representative images of flat-mounted retinas labeled with fluorescein-labeled Griffonia Simplicifolia Lectin 1 (GSL 1), isolectin B4 to examine vasoobliteration in animals exposed to 75% oxygen from P7 until P9. Animals were intravitreally injected at P7 with 100 μM of BH4 or vehicle (PBS steril 1×) and retinas analyzed at P9. Significant differences (p value) in the vasoobliterated area between vehicle and BH4 treatment after 48 h of hyperoxia are shown in the graphs; ***p < 0.0002 compared to hyperoxia. (PDF 292 kb) [file 12974_2017_955_MOESM7_ESM.pdf]
